# Constructing future scenarios as a tool to foster responsible research and innovation among future synthetic biologists

**DOI:** 10.1186/s40504-018-0082-1

**Published:** 2018-09-10

**Authors:** Afke Wieke Betten, Virgil Rerimassie, Jacqueline E. W. Broerse, Dirk Stemerding, Frank Kupper

**Affiliations:** 1 Athena Institute, VU University Amsterdam, De Boelelaan 1085, 1081 HV Amsterdam, the Netherlands; 2 Technical University Eindhoven (TU/e), Eindhoven, the Netherlands; 3 Rathenau Institute, The Hague, the Netherlands

**Keywords:** Responsible research and innovation, Ethics, Education, Synthetic biology, iGEM, Future scenarios, Learning

## Abstract

The emerging field of synthetic biology, the (re-)designing and construction of biological parts, devices and systems for useful purposes, may simultaneously resolve some issues and raise others. In order to develop applications robustly and in the public interest, it is important to organize reflexive strategies of assessment and engagement in early stages of development. Against this backdrop, initiatives related to the concept of Responsible Research and Innovation (RRI) have also appeared. This paper describes such an initiative: the construction of future scenarios to explore the plausibility and desirability of potential synthetic biology innovations. We guided teams of synthetic biology students who participated in the large international Genetically Engineered Machines (iGEM) competition, in constructing scenarios aimed at exploring the plausibility and desirability of potential synthetic biology innovations. In this paper we aim to examine to what extent, and how, constructing such future scenarios contributes to RRI. In order to do so, we conducted observations and interviews to understand what kind of learning and reflection was promoted by constructing the scenarios in terms of four dimensions, which are discussed prominently in the literature on RRI: anticipation, inclusion, reflexivity and responsiveness. While we focus on how constructing future scenarios can contribute to strengthening RRI at a project (and individual) level, we also consider how far our experiment may foster RRI in the iGEM competition in general, and perhaps even inspire constructive collaboration between ‘social scientists’ and ‘natural scientists’ in the context of larger scientific research programmes.

## Introduction

Synthetic biology can be understood as “the design and construction of new biological parts, devices and systems as well as the re-design of existing natural biological systems for useful purposes” (Roberts and Cranenburgh 2013, 1219). The field has attracted worldwide attention (see, for example, Church et al. 2014; Kaebnick et al. 2014; Si and Zhao, 2016). Some regard synthetic biology as a valuable tool in addressing important challenges in, for example, (public) health, natural resource management and energy security. At the same time, there are also concerns about potential risks as well as moral and social issues, for instance on the limits of ‘tinkering’ with life and nature or the socio-economic implications for developing countries (Rerimassie et al. 2016; ERASynBio 2014; IAP 2014).

Against this backdrop, several organizations made early assessments of potential ethical, legal and social aspects (ELSA) of synthetic biology and stimulated public engagement on the subject (see Rerimassie et al. 2016). In addition, initiatives related to the emerging concept of ‘Responsible Research and Innovation’ (RRI) have been playing a prominent role, particularly in Europe. One popular early definition of RRI appears in the 2013 policy document ‘Options for strengthening responsible research and innovation’ published by the European Commission. According to this definition, RRI “refers to the comprehensive approach of proceeding in research and innovation in ways that allow all stakeholders that are involved in the processes of research and innovation at an early stage (A) to obtain relevant knowledge on the consequences of the outcomes of their actions and on the range of options open to them and (B) to effectively evaluate both outcomes and options in terms of societal needs and moral values and (C) to use these considerations (under A and B) as functional requirements for design and development of new research, products and services” (EC 2013, 3)*.*

The term RRI was not introduced by the research field itself but in a top-down fashion by science policy-makers and various funding agencies mostly within the European Commission (first employed in the 7th Framework Programme in 2013). Regardless, RRI has attracted widespread academic attention, and accordingly has been – and continues to be – discussed and developed in academic publications and European-level projects (Burget et al. 2016; Zwart et al. 2014).

Without using the exact term, a discourse on responsible development of nanotechnology was already evident in the mid-2000s, according to Rip (2014). Indeed, the concept of RRI did not emerge out of the blue but builds upon earlier approaches and concepts on dealing with issues and questions related to scientific and technological development (Burget et al. 2016). In the field of the Life Sciences for example, *Bioethics* emerged in the 1980s as a plea to involve professional ethicists in addressing moral dilemmas in medicine and healthcare (Zwart et al. 2014). RRI also strongly draws on *Technology Assessment* (TA) and its various approaches (Zwart et al. 2014; Van Lente et al. 2017; Van Est 2017). Especially important is Constructive TA (CTA), which shifts the focus away from assessing impacts of new technologies to broadening design, development, and implementation processes. CTA builds on the concept of ‘co-evolution’ between science and society that was put forward by ‘science and technology studies’ (STS). Accordingly, CTA is one of the TA approaches in which stakeholders’ participation (and involvement of society in a broader sense) plays a crucial role (Schot and Rip 1997; Krabbenborg 2013). Another relevant approach is Real-time TA, which aims to integrate natural science and engineering investigations with social science and policy research from the outset (Guston and Sarewitz 2002).

Next to TA, *ELSA* (or ELSI in the US^1^), which stands for ethical, legal and social aspects of emerging sciences and technologies, emerged in the 1990s, serving as another important source of inspiration for RRI. The purpose of ELSA research was to provide a social and ethical component to science and technology development programmes (Zwart et al. 2014; Forsberg et al. 2018). Last, RRI is related to the concept of ‘*anticipatory governance’*, which is described as “a broad-based capacity extended through society that can act on a variety of inputs to manage emerging knowledge-based technologies while such management is still possible” (Guston 2014, 219). It is intended to motivate activities designed to build subsidiary capacities in foresight, engagement, and integration, as well as through their production ensemble (Barben et al. 2008; Guston 2014).

Furthermore, and in addition to building on these earlier concepts and approaches, RRI is strongly connected with ‘grand societal challenges’. Particularly in EU science policy, addressing such challenges has gained prominence. For instance, the Horizon 2020 programme defined tackling societal challenges as one of its main priorities (EC 2013). Orientation towards such grand societal challenges – or, more broadly speaking, desirable social goals – is reflected in several definitions and projects related to RRI (e.g. Von Schomberg 2013). As Zwart et al. note, the overall framing and explicit link to innovation and grand challenges distinguishes it from earlier approaches, such as TA and ELSA; the framing gives much more weight and urgency to the matter of channelling science to the common good. Finally, RRI can also be understood as a response to the dissatisfaction with earlier forms of interdisciplinary collaboration between natural and social scientists in socio-technical knowledge production and innovation. In such projects social scientists run the risk of being viewed as ‘nay-sayers’, the voice of negative criticism, which significantly constrains opportunities for bringing about changes in practice and for productive relations between natural and social scientists (Balmer et al. 2016).

Having now traced some of the roots, developments and ideas behind RRI, it is still not easy to come up with a clear-cut definition of RRI. In their 2016 literature review article, Burget et al. found no fewer than 235 RRI-related articles and concluded that there is still a lack of clarity concerning its definitions and dimensions. At the same time, they show that there is considerable interest in RRI. Having been promoted by the European Union (EU), national initiatives also emerged. For instance, the Dutch Science Council (NWO) initiated a programme on “Maatschappelijk verantwoord innoveren”, the Dutch version of RRI (van den Hoven et al. 2014) and in the UK the Engineering and Physical Sciences Research Council (ESPRC) applied RRI in the context of geo-engineering (Stilgoe 2016).

## RRI in the context of synthetic biology

Initiatives to promote RRI in the field of synthetic biology emerged as well. One of these initiatives was SYNENERGENE^2^ (2013–2017), a European project that aimed to contribute to RRI of synthetic biology by organizing activities that foster an open dialogue between a wide range of actors. SYNENERGENE organized multiple activities in order to mobilize a broad range of stakeholders to discuss what is socially desirable and how to collectively shape the development of synthetic biology accordingly (Albrecht et al. 2015; Stemerding et al. (in press)).^3^

In this paper, we reflect on one of these activities, where collaboration was sought with the community related to the international Genetically Engineered Machines (iGEM) competition. In the iGEM competition, teams of students use standardized genetic building blocks (BioBricks™) to design micro-organisms with novel and useful properties. The iGEM teams design, build and test their innovations over the course of the summer and gather at a ‘giant’ jamboree during the autumn to present their work. Because of its notable scale and scope^4^, the iGEM competition (and the iGEM community) is recognized as being very influential in the development of synthetic biology (Balmer and Bulpin 2013; Smolke 2009). One aspect of the competition, relevant to this paper, is the so-called ‘Human Practices’ work in which all teams engage. This work entails going ‘beyond the lab’; students have to imagine their projects in a real-life context and consider the social aspects of their research. Taking these ‘Human Practices’ into account is regarded as “crucial for building safe and sustainable projects that serve the public interest^”^.^5^

It is argued that the iGEM competition can be seen as an RRI laboratory (Stemerding 2015) and therefore provided us with an interesting space to learn about the further operationalization of the relatively new concept of RRI. In order to do so, we developed a two-step approach dedicated to imagining plausible and socially desirable synthetic biology futures, largely inspired by the concept of Real-time TA (Guston and Sarewitz 2002). First, from 2014 to 2016, we supported a number of iGEM teams in parts of their ‘Human Practices’ work, coaching them in constructing future scenarios aimed at examining the *plausibility* and *desirability* of their synthetic biology design. We define these activities as a *technological options-oriented approach* to RRI. Here, Real-time TA served as a stimulus to broaden technological design and development by increasing interaction and reflexivity, ‘opening up’ the laboratory to society (Doorn et al. 2014). As a follow-up activity, we deliberately shifted our focus to a *societal objectives-oriented* approach to RRI, engaging social stakeholders and scientists in a process of ‘mutual learning’ (Calvert and Frow 2013; Raman 2014; Selin et al. 2015) through interactive stakeholder workshops. Our approach thus explicitly sought to connect our activities with ‘grand societal challenges’. In order to ensure synergy between the two approaches we organized them around specific challenges, such as antibiotics resistance and renewable energy. Our Real-time TA approach thus involved two forms of future-oriented reflexivity. On the one hand, young synthetic biologists were challenged to critically examine technological promises and expectations by stepping into the wider world and engaging with social stakeholders. On the other, social stakeholders were invited to critically consider the nature of social problems, needs, values and purposes and the potential role of synthetic biology in responding to these challenges. Stemerding (in press). This paper focuses on our experiences in the first year of SYNENERGENE in which we guided seven teams in constructing future scenarios*.* This paper aims to examine to what extent constructing such future scenarios – and its accompanying activities and learning process – can be seen as a contribution to RRI. Accordingly, we formulated the following research question:

### To what extent, and in what ways, can constructing future scenarios contribute to RRI practices?

The paper is structured as follows: first, we discuss our hypothesis of how constructing future scenarios could contribute to RRI. Here we will also introduce the guidelines on constructing scenarios that we developed for the iGEM teams. Second, we elaborate on our research strategy and the analytical concepts we used for data analysis, followed by a discussion of the findings. Finally, we share lessons and draw conclusions. Here we consider how constructing future scenarios can contribute to RRI at a project level and iGEM in a broader sense, as well as the extent to which it may serve to inspire fruitful collaboration between the social sciences and natural sciences in the context of major research programmes.

## Imagining the future with application scenarios and techno-moral vignettes

As described by Lucivero (2012) it is challenging to integrate normative sensitivity in TA practices. Virtual imaginations of the feasibility and desirability of future innovations in which technical and social components are connected over time could enhance the integration of this ideal of normative sensitivity (Lucivero 2012; Selin 2011). In essence, imaginations of the future can be ‘tested’ in an anticipatory way, by integrating feedback from the external world in the virtual innovation (also called ‘the virtual prototype’). This can be seen as a way to conceive virtually of possible variations of future embedding of technologies, which can then be assessed in terms of plausibility and desirability (Selin 2011). In this sense future-making by means of scenarios can help to give a more concrete shape to variations of development trajectories, and to be able to analyse the explicit and implicit stories that feature in dealing with futures (Selin 2008, 2011).

In our project, we developed two sets of guidelines for constructing future scenarios: (1) to write *application scenarios* and (2) to create *techno-moral vignettes*. *Application scenarios* are empirically grounded speculations, based on our current understanding of the world, and describe how a particular innovation might be taken up in this context. *Techno-moral vignettes* are fictional with the aim of triggering imagination and reflecting on the desirability of a technology. These can use any genre, depicting future snapshots of wider social implications and value conflicts as ‘soft impacts’, in worlds where particular (synthetic biology) applications are imagined to have been widely adopted (Lucivero 2012; Swierstra and Molder 2012). An important challenge to note here is finding a balance between being too speculative or not speculative enough. As explained by Lucivero et al. (2011) the concept of plausibility is inherently intersubjective. This situated nature of judgments can be regarded as problematic, but, as Lucivero et al. (2011) argue, also allows us to explore and analyse the assumptions that characterize someone’s background and vision. The guidelines consisted of a variety of tools and exercises and relevant literature. For example, in the application scenario guidelines the students learn how to make and use stakeholder maps, personas, a product life-cycle analysis and filling in a business model canvas. In the guidelines for writing techno-moral vignettes the students learn to distinguish between hard and soft impacts and different argumentation patterns and how to incorporate these insights into their virtual prototype. During the iGEM project, the teams were coached by two STS researchers – authors AWB and VR – using the two sets of guidelines to give shape to the coaching process. In this, the coaches paid specific attention to those aspects that were not considered (enough) by the iGEM students or that the students were struggling with, for example in exploring soft impacts and alternative visions of their future scenarios. The coaching entailed (1) several (Skype) meetings in which activities, articles, and outputs were discussed, (2) contact via e-mail, and (3) (digital) feedback on draft versions of the future scenarios. Despite our coaching role, we should underline that – in the spirit of the iGEM competition – the starting point was that the students themselves bore the primary responsibility for the scenario work.

The real-time TA activities in which the teams engaged should also be seen as learning processes. While choices in the design of a technology reflect the choices of the innovator, increased awareness about broader issues that may come into play in future uses of the technology could in turn influence the internal considerations and values shaping the process of design (Poel 2013; Poel and Kroes 2014). In the language of the iGEM community, this internal purpose of scenario learning adds to *Integrated Human Practices*. In addition, the potential value of the scenarios is not limited to the iGEM team developing them. An important external purpose in human practices for iGEM teams is *Education and Public Engagement*, in which scenarios may serve as a medium for communication and debate with stakeholders or the broader public. Indeed, as SYNENERGENE partners, we have used such scenarios in theatrical debates, involving publics in discussions about futures of synthetic biology (van der Meij 2017).

## Research strategy and analytical concepts

For this study we examined the (learning) experiences of seven iGEM teams that we guided in their scenario work from May to October 2014. The teams consisted of 10–21 students with various disciplinary backgrounds, such as (molecular) biology, biotechnology, engineering, (bio)chemistry, bioinformatics, and computer science (BSc and MSc levels. (See Table 1 for details.)

**Table 1:** Participating teams

Team name / (university)	Topic	Guidance (author)	Interview with/
Bielefeld-CeBiTec (Bielefeld University)	The Transformers – From carbon dioxide to biofuel	AWB	1
Groningen (Rijksuniversiteit Groningen)	LactoAid – A smart bandage for burn wounds	VR	2
LMU-Munich (Ludwig-Maximilians-Universität München)	‘BaKillus’ – Engineering a pathogen-hunting microbe	VR	2
TU_Darmstadt (Technische Universität Darmstadt)	E. Grätzel – Solar BioEnergy	AWB	1
TU_Eindhoven (Eindhoven University of Technology)	Click Col – Expanding the chemical toolbox for bacteria	VR	2
TUFTS (Tufts University)	Ribosponge – Robust biofilm formation using a cyclic-di-GMP aptamer and investigating ethics and applications of engineered bacteriophage	VR	1
Wageningen UR (Wageningen University and Research)	BananaGuard – Biocontrol of Fusarium oxysporum using Pseudomonas putida	AWB	3

### Conceptual framework

To guide and structure the data collection and analysis, we used the conceptualization of RRI as comprising four dimensions: anticipation, inclusion, reflexivity, and responsiveness (Stilgoe et al. 2013). There were two main reasons to use this framework. First, given our interest in learning experiences, the framework proved valuable in terms of learning by research scientists, when Stilgoe (2016) put it to practice in the context of a geo-engineering project. Second, as Burget et al. (2016) point out, while the concept of RRI is discussed in different ways, these specific dimensions nevertheless appear prominently in the RRI literature. Accordingly, the framework matches the needs for our analysis, i.e. trying to understand the iGEM teams’ learning in terms of RRI, and the future operationalization of RRI. For each dimension we distilled key questions and indicators, set out in Table 2.

**Table 2:** Key indicators of RRI dimensions, summarized from Stilgoe et al. (2013)

Dimension	Key indicators
*Anticipation*	Showing the capacity to ask “what if”-questions. (What is likely? What is plausible?)
	Showing the capacity to think systemically
	Acknowledging the value of system thinking
	Explicitly recognizing complexities and co-evolution
*Inclusion*	Recognizing and acknowledging engagement beyond key stakeholders
	Diversifying the inputs to and delivery of governance
	Opening up framings of issues
	Recognizing engagement as a learning process
	Opening up discussion on future social worlds
*Reflexivity*	Being able to hold a mirror up to one’s own activities, commitments, and assumptions
	Being aware of the limits of (technical) knowledge
	Being mindful that a particular framing of an issue may not be universally held
	Scrutinizing value systems and theories that shape science and innovation
	Opening up alternatives
	Rethinking prevailing conceptions about the moral division of labour within science and innovation
	Recognizing wider moral responsibilities
*Responsiveness*	Acknowledging the need or possibility to change shape or direction in response to stakeholder and public values and changing circumstances
	Recognizing the limitations of knowledge and power
	Being able to situate the project in the wider political and economic landscape
	Showing the capacity to scrutinize science system elements and governance
	mechanisms (e.g. with regard to intellectual property regimes, and funding)
	Incorporating particular ethical values in their design

### Data collection and analysis

The results we present in this paper focus on the experiences of the students in doing the scenario work. The data were obtained from our observations during the guidance of the scenario work, document analysis, and semi-structured interviews with the teams a few weeks after the jamboree. The different methods informed one another and thus strengthened the subsequent analysis.

Observation: During the guidance of the scenario work, the supervisors made notes of their observations and experiences. During the iGEM jamboree that was held in October 2014, we hosted two workshops where the teams presented their scenario work and discussed theirs and others’ work. The workshops were audio-recorded to be able to contextualize and give more coherence to the narratives of the students’ experiences (see, for example, Emerson et al. 2001, 388).

Document analysis: We also used the outputs of the teams – their scenarios, vignettes, text on their wikis, and their presentations – as a means to further interpret the interviews and our observations.

Interviews: We held seven semi-structured exit interviews with in total 12 representatives of the seven teams. An interview guide was developed based on our experiences with the teams, their presentations during the workshop and current insights from RRI the literature, most notably the framework, as presented above. The objective was to guide the students in conveying their account of experiences related to RRI practices, using the guiding questions and context provided by our experiences and the workshops tapes to support the unfolding of narratives – as determined by the students themselves (see Galletta 2013, 48).

The interviews were transcribed verbatim and coded by authors AWB and VR. AWB and VR first thematically analysed the transcripts of the teams they guided independently (see, for example, Braun and Clarke 2006). The key indicators as presented in Table 2 were used to get a first understanding of the effects of the scenario work in terms of RRI. However, we adopted a bottom-up coding approach in which we stayed closer to our data to explore different interpretations of the four dimensions or sub-elements in the context of their (learning) experiences. After the first round of coding, AWB and VR reviewed each other’s analysis, and together they reflected on and refined the codes and themes. We then sub-clustered the results in themes to emphasize the specific elements of a certain dimension. As a final step we compared our interpretations to the conceptualization of Stilgoe et al. (2013).

## How did the scenario work contribute to RRI practices, according to iGEM students

In this section we describe our findings in terms of the four RRI dimensions and their indicators. Looking back at the scenario work, what did the iGEM teams experience and learn with respect to RRI? In order to illustrate our findings, we use quotes from the iGEM teams, mainly to highlight recurring themes. Occasionally, however, they illustrate a particular learning experience (limited to a specific team), which is mentioned if this is the case. First, we will give a brief impression of the scenarios developed by the iGEM teams.

### The scenarios developed by the iGEM teams

In terms of reporting towards SYNENERGENE, we asked the iGEM teams to provide a written description of their scenarios. Other than this, we did not impose any formal requirements of how they depicted their scenarios. All of the teams integrated their scenarios in their team websites (“wiki’s”) in the form of written texts, occasionally supplemented with infographics or other images. As intended, the parts relating to the application scenarios go beyond mere ‘scientifically oriented’ texts. In addition to describing the functionality and intended (future) use of their design, they focus on the broader social context of their project, such as outlining the (social) problem being addressed, business plan and regulatory context. All the teams did this, but the work of iGEM Wageningen^6^ and iGEM Tufts^7^ were particularly good examples. In the guidelines for the construction of techno-moral vignettes we provided the teams with examples of vignettes previously developed in the “SynBio scenarios” project of the Dutch Rathenau Institute^8^, and the techno-moral vignettes made by the teams were inspired by these examples. Teams often created short stories focusing on a moment in the future in which their innovation had an impact on society. They used the insights from their application scenarios as input. Interesting examples here are the vignettes prepared by iGEM Darmstadt^9^ and iGEM LMU-Munich.^10^ Clearly, the outputs of the different teams varied but they were all successful in developing perceptive and informative scenarios and vignettes. Although outputs can be judged in several ways, we consider ‘success’ not to be a measurable or objective valuation of their work, but rather focus on how the work contributed to their learning process.

### Anticipation

Stilgoe et al. (2013) describe anticipation as the capacity to recognize complexities and think systemically, thereby generating a socially robust agenda for (risk) research and innovation. During the interviews, students described three experiences and learning moments indicative of anticipation: (1) understanding the project as an iterative process of inquiry, (2) seeing the bigger picture, and (3) considering ‘the outer world’ early in the process.

#### Understanding the project as an iterative process of inquiry

One thing that stood out in the students’ experiences was how the scenario work helped them in creating an iterative process of inquiry. The first example below shows how students organized multiple moments of reflection by going back to their scenario several times.

*We shared them [the possible scenarios] and had someone else read them. [...] We had several rounds of feedback, you do A, I do B, then turn it around. Read it again and add your suggestions.* (interview, TU/e).

Connected to this idea of ‘going back and forth’ was what students described as the obligation to tie up loose ends. As one student says:

*Writing it down is a structured way to really bring it together [...] then you will notice the holes and think “I need to figure this out”.* (interview, WUR).

These examples reflect insights into the complexities of technological development; the idea that in trying to make predictions you have to navigate between the outer world and your innovation.

#### Seeing the bigger picture

Another point that students often voiced related to how the construction of scenarios helped them in ‘seeing the bigger picture’. Most students recognized this potential in terms of being able to tell a coherent and ‘honest’ story. They explicitly mentioned the wish to be honest about the impact of their innovations, which was not always easy in the context of a competition. As one student described:

*Sometimes it can be more about selling [...] and you are not always honest in that, because you also want to win.* (workshop at jamboree, TU Darmstadt).

Furthermore, the process helped in recognizing the interwoven technical and social elements of their innovation, beyond deficit ideas of the public.

*I didn’t expect it to be so helpful, there was so much more to it [the technological part], we learn about safety and security, but now [...] also about things like how someone’s life might change, all kinds of things related to work and culture.* (workshop at jamboree, Bielefeld-CeBiTec).

#### Considering ‘the outer world’ early in the process

A third indicator of anticipation relates to the notion of time in reacting to things from ‘the outer world’.

*We saw this [SYNENERGENE] project as a way to understand the risks better, and to be able to counteract them – also in case of questions of course.* (interview, TU/e).

This quote shows that the scenario work helped them in answering questions and considering concerns before they were going to be asked about it. This sense of preparedness was also beneficial to the students because it helped to motivate them. As another student puts it:

*It was a great feeling to feel like I was, or we were, in charge, and that if something would come up we could easily deal with it, questions, or things that needed to be [...] adjusted, [...].* (personal communication, Bielefeld-CeBiTec).

### Inclusion

Following Stilgoe et al. (2013), inclusion should be seen as a learning process in which new forms of deliberation go beyond engagement with key stakeholders to open up discussion of future social worlds. From our results it becomes clear that the scenario work enhanced the students’ understanding of this more nuanced perspective of inclusion, but that there is room for improvement – especially with regard to its aim of critically interrogating the ‘social constitutions’ inherent in technological options. Students described two experiences and learning moments indicative of inclusion: (1) being aware that inclusion is not an end in itself, and (2) seeing inclusion as a learning exercise.

#### Being aware that inclusion is not an end in itself

Most students acknowledged that inclusion should not be seen as an end in itself. Interestingly, in one case this insight led to not inviting any members of the public during the project. This was not because they did not want or did not see any potential to do so: it was more the question of the combination of time issues and the desire to do something only if it was in fact meaningful.

*I mean, only if your project is about education or something, then it makes sense to go to a school, but other than that it makes no sense [..] For our project we just did not have such a group, and we didn’t have the material ready early enough to reach out to wider audiences.* (interview, TU Darmstadt).

For this team, the scenario work supported learning about inclusion that was very meaningful to them.

#### Seeing inclusion as a learning exercise

The idea of inclusion as a learning exercise entails two elements: the first is that the process should organize feedback into the technological project, and the second is that it should open up framings of issues and future social worlds. The first element is something most students reflected upon; they used the scenarios and vignettes explicitly in organizing feedback into the project. Many emphasized how pleased they were with how they could connect their explorations to their other (more technical) work.

*Initially, we went to the hospital with an educational mind set [...] because of SYNENERGENE we really went looking for the weaknesses in our product. [...] We went back to the hospital and thought “we want to know more about the ethical aspects”.* (interview, RUG).

This quote shows how not only they managed to engage with stakeholders they would not have done otherwise, but it also reflects insights into plural perspectives and appreciation of other types of knowledge (linking also to the dimension of reflexivity).

The idea that whatever comes out of engagement practices should find its way back into the project is not new at iGEM (it is on the list of judgment criteria) but many students acknowledge that this was not easy. They can imagine how this would work easily in cases of a clear target group (e.g. when the project is about developing a bedside diagnostic tool and they can do interviews with patients about their needs and ideas). Considering this difficulty, the construction of scenarios helped the students to broaden their perception of who can or should be included in an innovation project. As this student refers to the creation of a persona (which was one of the tools/exercises in the guidelines):

*It really helped to think about this man, and where he lived, and the life he was living [...] our product came to life sort of, [...] if you think longer, there are so many people affected eventually by something.* (interview, TU Darmstadt).

It was an explicit part of the second set of guidelines: trying to think about how others (end-users, patients, people living in a certain part of the world, parents, farmers, etc.) would look at the problem they were dealing with. Although it remained complex to tie these insights to inclusionary practices, many students explicitly described how their experiences led to an increased understanding of the plurality of framings.

### Reflexivity: Moral awareness

According to Stilgoe et al. (2013), reflexivity entails, for instance, being able to hold a mirror up to one’s own activities, commitments, and assumptions, as well as recognizing wider moral responsibilities. We found that working on the future scenarios strengthened the iGEM teams’ reflexivity. All teams expressed – in different ways – that it contributed to a broader sense of *moral awareness*. This applies to the teams in general (and thus the project), but even more so, at a personal level.

First, the scenario work triggered a broader moral awareness. While it may have taken some time before this manifested itself, it was found to be valuable as well as fun. The iGEM team from RUG found themselves challenged to look beyond ‘typical’ risk-related questions and found this inspiring. It also led to questioning the position of scientists in society, as expressed by two members of the iGEM team from LMU-Munich. When discussing the work on the techno-moral vignettes during their exit interview they remarked:

*Scientists always say: it’s not my business. I just do it because its science and it brings us further. But what is good science? It brought us more in the direction to really consider it. Could there be a better way? Is it really good what we do?* (interview, LMU-Munich).

The other team member continued:

*I think today science is very often very short-sighted. I mean theoretically think of the next set of results that he can publish. I think that really thinking for a second and extrapolating to the future can be really helpful to shape your present work now.* (interview, LMU-Munich).

Another student described how he thought that the scenario work experience helped him to develop a more critical lens:

*These are questions that really matter in the context of SynBio. [...] It’s really a matter of looking at things differently, from a critical stance, and that is something I developed, which will remain, I’m sure.* (interview, WUR).

These quotes demonstrate how working on the scenarios challenged the participants to reflect on their role as scientists in society and enabled them to consider wider social perspectives. Interestingly, working on the scenarios turned out to be helpful in triggering such reflexivity in the context of basic research. The iGEM TU/e team developed a system called ‘Click Coli’, which would allow one to ‘click’ different types of molecules on top of *E.coli,* such as coatings. Working on future scenarios helped the team to identify real-world applications in which their basic part could play an important role (iGEM TU/e, 2014). In their exit interview one of its team members noted that:

*You must keep an eye on an eventual goal. You cannot do basic research only for basic research purposes. By working on techno-moral vignettes you make sure that a team doing basic research considers concrete applications.* (interview, TU/e).

A representative of TUFTS drew the same conclusion:

*You are focused very specifically on the research. You rarely get to see that overarching picture. iGEM helps and I think SYNENERGENE helped more, because it gave you those guidelines and required you to do so.* (interview, TUFTS).

### Responsiveness

The final RRI dimension we consider is ‘responsiveness’: a capacity to change shape or direction in response to stakeholder and public values and changing circumstances (Stilgoe et al. 2013). We observed two ways in which the scenario work strengthened the teams’ ‘responsiveness’: by opening up their design to insights from the real world and by identifying meaningful courses of action.

#### Opening up the design

One of the teams, the iGEM team from TU Darmstadt, made changes to their design partly as a result of the scenario work. The team aimed to address problems concerning access to electricity in African countries. In their application scenario they describe how rural areas face a lack of access to (stable) power grids. Against this backdrop and given the limitations of currently available solutions, they argued that an off-grid system with low maintenance costs would be best suited to local conditions and population density to address this issue. To this end, they intended to engineer *E. coli* to produce a dye to be used in so-called ‘*Grätzel cells*’*.* These are electrochemical solar cells that use a dye instead of a silica semiconductor material for the absorption of light. When they took Senegal as an example country – chosen for its difficult socio-economic and environmental conditions – they concluded that their product could contribute most in places other than where it would be manufactured, and that the product should be suitable for downstream processing. In the production of dye-sensitized solar cells it is common to use *anthocyanins*: pigments that are soluble in water. In order to facilitate easier shipping and reduce costs, however, the product should preferably be in powder form. For this reason, they changed their chosen dye from naringenin to pelargonidin, which is an *anthocyanidin* – the sugar-free counterpart of anthocyanins – that ensures extraction with organic solvents, which makes it easier to get the product into powder form. In order to do so, the team had to redesign the pathway of their engineered *E. coli* to produce this type of dye (iGEM TU Darmstadt 2014 policy and practices).

#### Identifying meaningful actions

For other teams, working on the scenarios did not lead to changes in the design as such, but it nevertheless inspired several actions. The iGEM RUG team developed ‘LactoAid’, a smart band aid to treat burn wounds and prevent infections. The objective was to develop this into a commercial product. Discussing how the scenario work affected their project, one of the team members remarked:

*While working on the application scenario we considered the implementation of our product and learned that we’d ought to start in a hospital setting first. This is a heavily regulated environment, which at the same time would allow the implementation of the band aid. You cannot expect to have it in the drug store immediately.* (interview, RUG).

Working on the scenarios thus increased this team’s knowledge on how to implement their product. First, they targeted implementation in hospitals (where the band aid certainly would be valuable, according to stakeholder interviews). Later, the team aimed to target commercialization in stores, but only after the band aid had already been used in a controlled setting. Working on the scenarios thus contributed to the alteration and optimization of their implementation scheme.

### Other lessons learned

Besides our insights into how, in this project, scenario work contributed to dimensions of RRI, we would like to share two other connected lessons: (1) the importance of writing and guidance, and (2) the importance of a sense of meaningfulness.

#### The importance of writing and guidance

When asked if the guidelines could be of use without having to construct scenarios and vignettes the students unanimously responded that the writing process was an essential part, as opposed to merely using a checklist. Several teams were convinced that without the actual writing of scenarios, crucial (moral) questions would not have emerged:

*Because of the story element you get to the ethical things. Ethical questions do not emerge through scientific texts. By evoking empathic moments with a character you get to imagine the potential ethical consequences.* (interview, TU/e).

In addition, many students commented on the positive effects of the collaboration with us as STS researchers. Most students found the multiple Skype conversations and feedback rounds essential. Some students remarked that without guidance they would not have given the scenario work as much attention, because they learned the added value only later in the course of the project. Students also said that at the beginning of the collaboration (on reading parts of the guidelines), they were concerned about their outputs not being up to standard or as expected by us. Even though we sought to emphasize that it was not the point to create perfect scenarios and that the value of (making) the scenarios lay in other (often unexpected) things, the undefined nature of possible outputs led to some concerns. Especially in an educational context (see below) these points might hamper the learning process in profound ways.

#### Meaningful human practices

Students often compared the scenario work to courses on social aspects of technology they had previously attended or to previous iGEM human practices work and emphasized how it was more meaningful for their projects and hence for them. Most students described moments where they felt *“it clicked”*, or *“it all came together”*, or *“finally made sense”*. It must be noted that most of these moments were rather late in the process, which can be a problematic point – certainly without guidance. Also, it has to be said that these experiences describe moments where a lot happened at the same time, and it is difficult to pinpoint what causes a moment of success and what constitutes ‘meaningfulness’.

## Conclusion and discussion

In recent years RRI has emerged as a novel approach in dealing with questions and issues relating to scientific and technological development, building on earlier traditions, such as Bioethics, ELSA and Technology Assessment. In the context of SYNENERGENE we aimed to operationalize RRI along two forms of future-oriented reflexivity. First, by following a *technological options-oriented approach*, focused on iGEM teams that critically examined their innovation through the construction of future scenarios. Second, by following a *societal objectives-oriented approach,* stakeholders were invited (in a subsequent step) to discuss the nature of social problems, needs, values and purposes and the potential role of synthetic biology herein. This paper dealt specially with the former. In this concluding section, we will first consider whether the scenario work – including collaboration with us as ‘STS coaches’ – contributed to RRI on the ‘micro scale’ of the iGEM projects. In addition, we compare our findings to the conceptualization of these dimensions as described by Stilgoe et al. (2013). Next, we discuss the limitations of our study and consider to what extent our experiment can contribute to fostering RRI in the broader context of the iGEM competition. Finally, we consider – in all modesty – whether it can serve as an inspiration for constructive future collaboration between ‘social scientists’ and ‘natural scientists’ in the context of larger scientific research programmes.

### Contribution of scenario work to the practice and conceptualization of RRI

Based on the results of our experiment, we conclude that the scenario work contributed to the operationalization of RRI in the context of the projects of the iGEM teams, (and thus, in the terminology of the competition, to meaningful human practices work). Overall, the results suggest a positive impact on the four dimensions of RRI: anticipation, inclusion, reflexivity, and responsiveness. Here, we will briefly discuss important findings related to each dimension, followed by a description of two interconnected ways in which we saw that that the scenario work contributed to RRI.

First, as described by Stilgoe et al. (2013), *anticipation* revolved around the development of the capacity to think systemically. In order to anticipate one should be able to recognize co-evolutionary complexities, for instance by understanding the dynamics of promises and expectations that shape development (Borup et al. 2006). Our results suggest, however, that even though students often described situations of ‘seeing the bigger picture’, we would not say that a systems thinking approach was truly adopted. We did see that students deployed a strategy of going back and forth between their innovation and the ‘real world’, which, we would argue, is a step in the direction of being anticipative: it acknowledges that such an iterative process is vital, but it is not necessarily built on unravelling underlying dynamics that shape innovation.

As emphasized by Stilgoe et al. (2013), one of the key elements of the function of *inclusion* is that it should open up discussion on future social worlds. It is explicitly not (only) about engagement of stakeholders, and the realization that engagement for its own sake is not inclusive should be key in this. Our results suggest that students did become more aware of this notion, partly because they were already seeking to find ways for ‘more meaningful’ human practices. With regard to deficit understanding of the public, our results indicate mixed effects: students made attempts to emphasize how the public’s perspectives should be taken into account, but still focused quite a lot on risk and knowledge communication in this regard.

Stilgoe et al. (2013) provide a threefold conceptualization of *reflexivity* that entails: the ability to hold a mirror to one’s own activities, commitments, and assumptions, being aware of the limits of (technical) knowledge and being mindful that a particular framing of an issue may not be universally held. It also means that prevailing concepts about theories that shape science and technology development and about moral division of labour within innovation should be opened up for enquiry. In our study, we observed that the scenario work facilitated awareness of other values and expertise, i.e. moral awareness. This shift towards reflection on one’s own background and the underlying value systems is what Schuurbiers (2011) refers to as ‘second-order reflexivity’ where values also become object of study. Furthermore, we observed a shift in focus from responsibility in terms of safety and security towards a focus on responsibility in terms of the role of science. We did not, however, see an increased awareness in terms of the limits of knowledge as such. Students did acknowledge other perspectives as being important (see above) but this was more about overcoming issues of acceptance and possible mismatches than a sign or reflexivity with regard to the limits of technical knowledge.

Last, Stilgoe et al. (2013) describe *responsiveness* as an acknowledgement of the need to shape innovation trajectories in response to public values and changing circumstances. Similar to the dimensions of anticipation this requires scrutinizing the systems of power and governance that shape innovation processes. We saw that students were looking for ways to adapt their design based on insights from the real world. In that sense they were open to changing their original plans, but this was more in terms of broadening their scope of looking for information and input for their project, as well as identifying meaningful courses of action to move forward. Similar to what Smith et al. (2017) describe, we saw that students tend to understand responsiveness in terms of making an appropriate connection between their innovation and the context of its use. Here, the fact that the students have to create a specific technological output influences the possible level of responsiveness, and it is challenging to find a balance between creating something tangible that is also open at the same time (Smith et al. 2017), especially in the context of a competition. Accordingly, albeit beneficial, the teams’ responsiveness was not much based on (the acknowledgement of) responding to public values.

We would at this point like to describe two factors of the scenario work (as implemented in the project) which enabled the outcomes on the different RRI dimensions. This is related to what Stilgoe et al. (2013) describe as the blurred lines between dimensions, which is important for integration and mutual reinforcing. First, the specific link between the scenario work and each individual innovation project made engagement more meaningful for the students; they took greater pleasure in doing it as they saw more added value in comparison to other (previously experienced) work into social dimensions of technology development. This level of *real* investment can be essential for all dimensions of RRI since it stimulates motivation and enhances participation. Second, related to this idea of specificity was that the scenario work added coherence to their overall project. This also contributed to seeing an added value in this kind of work, but it also organized integration between different dimensions, such as going back and forth between the written scenario and possible responses of the public towards their scenario.

In conclusion, our results suggest that several elements of RRI dimensions were enhanced by the scenario work. We do however, realize that our guidance as ‘STS coaches’ played an important role. In the spirit of the iGEM competition, the teams themselves were responsible for the process and our involvement was therefore limited. At the same time, we note that, for instance, monitoring whether aspects mentioned in the guide were considered, clarifying ideas and providing examples is still some form of intervention. It would be fair to assume that without this interaction – modest as it may have been – the results and learning experiences would have been different.

### Limitations of this study

Finally, we wish to discuss some limitations of this study. First, we would like to reflect on our own role in guiding the teams in their scenario work. As the teams were guided by different coaches – some by AWB and others by VR - this might have affected the scenario work and comparison of results. To minimize the potentially negative impact, the authors consulted with each other regularly throughout the process, designed and facilitated the workshops together, and jointly analysed the data. The second limitation relates to the generalizability of this study. Because the students participated voluntarily in the collaboration, and they were responsible for the human practices part for their study, the results might not be the same for a different group of students. That having been said, it was not our aim to quantify how well the scenario work contributed to RRI, but rather *in what ways*.

### Looking ahead

We conclude by considering whether our experiment could contribute to fostering RRI in the iGEM competition in general and perhaps even inspire constructive collaboration between ‘social scientists’ and ‘natural scientists’ in the context of larger scientific research programmes.

First, we note that the iGEM competition is a very specific context in which our experiment took place. Against this backdrop, we want to highlight the following positive aspect that came out of the interviews with the students with regard to the constructing of future scenarios – that of candour. Being in a tough competition like iGEM can have personal consequences (see Smolke 2009), and thus may lead to masking failures or over-selling or hyping up results that constitutes an issue of relevance to the broader synthetic biology community (see, for example, Frow 2013; Pardo Avellaneda and Hagen 2016). According to the students in our study, the scenario work opened up the possibility to be more candid because they had more to communicate about. In other words, because they already had a grounded story, they did not have to make one up. As also suggested by Hartley et al. (2016), in identifying key features of responsible governance of biotechnology, we feel that these insights from students’ scenario work could be inspiring with regard to dynamics regarding issues of transparency and promises in communication and governance. Furthermore, we are fully aware that we were able to work with only a very small number of the iGEM teams that participated, and we learned that coaching was actually identified as one of the success factors. In the future application of the scenario work in iGEM this approach is not sustainable, however. The question is, therefore, how to make the guidelines usable without guidance of a ‘STS coach’. The initial guidelines were presented as successive steps in the form of a written protocol. Knowing that the SYNENERGENE project was coming to an end, and hoping to make a lasting contribution, the initial guidelines were developed into a more flexible and attractive, interactive web-based tool, publicly available on the iGEM website as the “iGEMer’s Guide to the Future” (https://live.flatland.agency/12290417/rathenau-igem/).^11^ Inspired by the feedback from the iGEM teams that worked with the initial guidelines, it was designed in a modular structure in which all – or just a few – exercises and tools can be used in various sequences. In addition, it was designed in such a way that the need for an STS coach was (hopefully) limited.

Finally, the outcomes of this study also suggest some directions for collaborations between the natural and the social sciences (and humanities). As mentioned before, RRI can also be understood as a response to the growing dissatisfaction with earlier forms of interdisciplinary collaboration between natural and social scientists in socio-technical knowledge production and innovation. In such projects social scientists run the risk of being positioned as ‘nay-sayers’ (Balmer et al. 2016). Accordingly, there have been recent attempts to organize more constructive interdisciplinary cooperation on a *programme level* (see Forsberg et al. 2018). One such example was the institutionalization of Risk Analysis and Technology Assessment (RATA) in NanoNextNL, a large-scale Dutch national research and technology programme for micro- and nanotechnology (see Wezel et al. 2018). We argue that interdisciplinary collaboration based around the construction of future scenarios may contribute to fostering further and inspiring integration of the social and natural sciences in such programmes and thus to the operationalization of RRI.

## Footnotes

1 As Zwart et al. (2014) explain, in the US the term implications (hence: ‘I’ was used), while later, in the EU framework, the term ‘aspects’ (‘A’) was used. Generally, this was seen as an effort to broaden the scope of the research, i.e. to avoid the flawed linearity implied by ‘implications’ and to launch a European alternative to the US version.

2 Funded by the European Commission’s FP7 Science in Society Work Programme.

3 The Rathenau Instituut, the Dutch office for Technology Assessment and Science System Assessment, (the employer of VR and DS) promoted political and social engagement with synthetic biology early on in its development (Rerimassie 2016). The concept of RRI offered an addditional and inspiring perspective for the institute to broaden its range of engagement activities regarding synthetic biology (Stemerding in press). The same applied to the Athena Instituut (employer of AWB, FK and JEWB) whose aim is to study and design constructive interfaces between science and society. For these reasons, the participation of both institutes in SYNENERGENE was appealing.

4 In 2014, 245 teams with a total of 4515 students participated in the competition.

5
http://2017.igem.org/Human_Practices

6
http://2014.igem.org/Team:Wageningen_UR/outreach/synenergene
http://2014.igem.org/Team:Tufts/app_scenarios.

7
http://2014.igem.org/Team:Tufts/app_scenarios

8 http://www.fi.uu.nl/toepassingen/28325/.

9 http://2014.igem.org/Team:TU_Darmstadt/PolicyandPractices/Techno-moralVignette.

10 http://2014.igem.org/Team:LMU-Munich/Rathenau.

11 The guide can be found on iGEM’s website here: http://2017.igem.org/Human_Practices/Resources.
